# Standardized *Sanguisorba officinalis* L. Extract Inhibits Adipogenesis and Promotes Thermogenesis via Reducing Oxidative Stress

**DOI:** 10.3390/antiox12040882

**Published:** 2023-04-04

**Authors:** Yulong Zheng, So-Yeon Lee, Yeji Lee, Tae-Kyeong Lee, Ji Eun Kim, Tae Hyeon Kim, Il-Jun Kang

**Affiliations:** 1 Department of Food Science and Nutrition & the Korean Institute of Nutrition, Hallym University, Chuncheon 24252, Republic of Korea; 2 Ju Yeong NS Co., Ltd., Seoul 05854, Republic of Korea

**Keywords:** *Sanguisorba officinalis* L. extract, obesity, reactive oxygen species, oxidative stress, antioxidant enzymes, AMPK pathway, thermogenesis

## Abstract

Obesity produces many health problems, including systemic oxidative stress. This study comprehensively investigated the effects of *Sanguisorba officinalis* L. extract (SO) as an antioxidant on abnormal lipid accumulation and oxidative stress in 3T3-L1 adipocytes and high-fat diet (HFD)-induced obese mice (*n* = 48). We evaluated the anti-adipogenic and antioxidant effects of SO on 3T3-L1 by cell viability, Oil red O staining, and NBT assays. The ameliorative effects of SO in HFD-induced C57BL/6J mice were investigated by measuring body weight, serum lipids, adipocyte size, hepatic steatosis, AMPK pathway-related proteins, and thermogenic factors. In addition, the effect of SO on oxidative stress in obese mice was evaluated by the activity of antioxidant enzymes and the production of lipid peroxidation products and ROS production in adipose tissue. We found that SO dose-dependently decreased lipid accumulation and ROS production in 3T3-L1 adipocytes. In C57BL/6J obese mice, SO (above 200 mg/kg) attenuated the HFD-induced gain in body weight and white adipose tissue (WAT) weight without affecting appetite. SO also decreased serum glucose, lipid, and leptin levels and attenuated adipocyte hypertrophy and hepatic steatosis. Furthermore, SO increased the expression of SOD1 and SOD2 in WAT, decreased ROS and lipid peroxides, and activated the AMPK pathway and thermogenic factors. In summary, SO reduces oxidative stress in adipose tissue by increasing antioxidant enzyme activity and improves obesity symptoms through AMPK-pathway-regulated energy metabolism and mitochondrial respiratory thermogenesis.

## 1. Introduction

Obesity has long been a worldwide public health problem [1]. Obesity makes the body bloated and brings inconvenience to daily life. More importantly, excessive obesity can make people vulnerable to atherosclerosis, heart disease, diabetes, hypertension, and other metabolic syndromes [2]. Adipose tissue is the main organ that regulates the overall energy homeostasis of the organism [3]. It stores excess energy in triglyceride (TG) form broken down to nourish other tissues during low energy availability [4]. Therefore, the fundamental reason for obesity is a persistent imbalance between energy intake and consumption.

Adipose tissue is mainly divided into two tissue types with different characteristics: white adipose tissue (WAT) and brown adipose tissue (BAT) [5]. WAT is the primary energy storage organ converting excess energy into TG for accumulation. In contrast, BAT is mitochondrially enriched and converts fatty acids to heat via uncoupling protein-1 (UCP-1)-mediated mitochondrial respiration [6]. Recent studies have found a beige adipose tissue that is an intermediate between WAT and BAT [7]. Beige adipose tissue shares characteristics similar to WAT, but its gene expression pattern tends toward BAT and it also has thermogenic effects [8]. Given the inducibility of beige adipose tissue, inducing gene expression in WAT to beige adipose tissue has emerged as a novel strategy for treating obesity [9].

Another problem with obesity is persistent oxidative stress. Obesity-induced high concentrations of free fatty acids circulating in the bloodstream activate the nicotinamide adenine dinucleotide phosphate (NADPH) oxidase pathway, and abnormal accumulation of lipids disrupts the antioxidant defense system of adipocytes [10]. These consequences initiate a chain reaction that leads to a rise in reactive oxygen species (ROS) levels in adipocytes and systemic oxidative stress. Excessive ROS accumulation raises proinflammatory factors [11] and reduces insulin sensitivity [12], causing adipokine disturbance [10] and mitochondrial dysfunction [13]. Therefore, natural products with antioxidant properties have the potential to improve obesity symptoms [14]. Korean traditional herbs such as mulberry leaf [15] and red ginseng [16] have been reported to alleviate oxidative stress and improve metabolic syndrome.

*Sanguisorba officinalis* L. (SO) is widely distributed as a medicinal natural product in China, Korea, and Japan and is well known for its antioxidant properties [17,18]. SO has been proven to have anti-inflammatory [19], antibacterial [20], antitumor [21], and hypoglycemic [22] physiological activities in addition to its antioxidant effects. Traditionally, SO is not only an edible plant but has also been used to treat ailments such as duodenal ulcers, diarrhea, hemorrhoids, and varicose veins [23]. This research aimed to assess the antioxidant capacity of SO in adipocytes and investigate its potential to improve obesity symptoms through thermogenesis.

## 2. Materials and Methods

### 2.1. Reagents

Ethanol (EtOH) was acquired from Daehan Ethanol life (Hwasung, Republic of Korea). Acetonitrile (ACN), methanol (MeOH), and isopropanol were acquired from J.T. Baker Chemical (Phillipsburg, NJ, USA). Dulbecco’s modified Eagle’s medium (DMEM) was acquired from Welgene (Daegu, Republic of Korea). WST-1 cell viability assay kit was acquired from Dogenbio (Seoul, Republic of Korea). Bovine calf serum (BCS), fetal bovine serum (FBS), penicillin/streptomycin (P/S), insulin, RIPA buffer, and BCA protein assay kit were acquired from Thermo Fisher Scientific (Waltham, MA, USA). Quercitrin, dimethyl sulfoxide (DMSO), dexamethasone (Dex), 3-isobutyl-1-methylxanthine (IBMX), nitro blue tetrazolium (NBT), dihydroethidium (DHE) 3,3′-diaminobenzidine tetrahydrochloride (DAB), Oil Red O (ORO), 2-methyl-2-butanol, and 2,2,2-tribromoethanol (avertin) were acquired from Sigma-Aldrich (St. Louis, MO, USA). The primary and secondary antibodies utilized in this research were acquired from Cell Signaling Technology (Danvers, MA, USA), Santa Cruz Biotechnology (Dallas, TX, USA), and Abcam (Cambridge, UK).

### 2.2. Sample Preparation

*Sanguisorba officinalis* L. was provided by JuYeong NS Company (Seoul, Republic of Korea). Dried leaves and stems were extracted twice with 54% EtOH at 73 °C under reflux for 8 h. The extract was condensed using a vacuum and converted into a powder sample by spray drying. The obtained *Sanguisorba officinalis* L. extract (SO) was stored in a desiccator.

### 2.3. Sample Standardization

Samples were standardized by HPLC analysis of quercitrin content in the extracts. The Agilent 1260 system (Agilent Technologies, Waldbronn, Germany) consists of a quaternary pump (G311), standard autosampler (G1329B), column oven (G1316A), and diode array detector (G4212B) that detected an output signal at a wavelength of 254 nm which was used to conduct HPLC analysis. Extract (1 g) was weighed into a 100 mL volumetric flask and dissolved by ultrasonication with 80 mL 50% MeOH for 20 min. Then, the same solvent was applied to the line of the flask and filtered with a 0.45 μm syringe filter (Whatman, Marlborough, MA, USA). In addition, quercitrin standards were dissolved in 50% MeOH, sonicated, and filtered. The solution was then diluted according to concentration. SO was chromatographically separated using a Kinetex C_8_ column (4.6 × 250 mm, 5 μm, Phenomenex Inc., Torrace, CA, USA) at a column temperature of 30 °C. The solvent system for analyzing SO comprised phase A (water with 0.1% formic acid) and phase B (ACN with 0.1% formic acid) flowing at a 1.0 mL/min rate. The conditions for gradient elution involved 90% A at 0–5 min, 75% A from 5–30 min, 90% A from 30–35 min, and 90% A at 40 min. HPLC analysis was repeated 3 times at different periods to measure the quercitrin content in the extract.

### 2.4. Cell Culture and Differentiation

The 3T3-L1 preadipocytes (American Type Culture Collection; Manassas, VA, USA) were grown in DMEM supplemented with 10% BCS and 1% P/S at 37 °C and 5% CO_2_ level. Cells were inoculated into 24-well plates at 5 × 10^4^ cells/well density and allowed to confluence. The differentiation medium (MDI; DMEM containing 10% FBS, 1% P/S, 0.5 mM IBMX, 1 μM Dex, and 10 μg/mL insulin) was replaced two days after the cells were 100% confluent and recorded as day 0. The differentiation medium without IBMX and Dex on was changed on day 2 and the differentiation medium containing only 10% FBS and 1% P/S on day 4. After that, the medium was changed every two days until day 8. Cells were treated with 100–400 μg/mL of SO and 200 μg/mL of *Garcinia cambogia* water extract (GC) at each medium change.

### 2.5. Cell Viability Assay

The viability of 3T3-L1 preadipocytes and adipocytes was measured using the WST-1 assay kit. Preadipocytes were seeded in 24-well plates at a density of 5 × 10^4^ cells/well and treated with SO (100–400 μg/mL) for 24 h at 100% confluency, or adipocytes’ cell viability was measured on day 8 of induced differentiation. After incubation, 500 μL of medium (containing 20 μL WST-1) was replaced in each well. Following 2 h later, the medium was moved to 96-well plates, and the optical density was quantified at 450 nm by a UV-visible spectrophotometer (Multiskan FC; Thermo Fisher Scientific, Waltham, MA, USA).

### 2.6. ORO Staining and NBT Assay

Adipocytes differentiated to day 8 were immobilized in 4% paraformaldehyde (Biosesang, Seongnam, Republic of Korea) for 40 min at room temperature. Subsequently, the cells were rinsed with 60% isopropanol and stained with ORO solution for 20 min at room temperature. The dyed cells were visualized through a microscope and photographed (ECLIPSE Ni-U; Nikon, Melville, NY, USA). The dried cells were redissolved in 100% isopropanol, and the UV-visible spectrophotometer was used to measure the absorbance at 520 nm. ROS accumulation in adipocytes was measured using the NBT assay. Differentiated adipocytes were incubated with 0.2% NBT solution for 90 min. Following cell drying, 300 μL of DMSO (DMSO:1 N KOH = 7:3) was introduced into each well and eluted for 10 min on a shaking device. Then, 300 μL of distilled water was introduced into each well, and the UV-visible spectrophotometer was utilized to quantify the absorbance at 570 nm.

### 2.7. Animal Experiment

Male C57BL/6J mice with a body weight ranging from 18–20 g and aged 5 weeks were obtained from Central Laboratory Animal Inc. (Seoul, Republic of Korea). The mice were housed in a 12-h cycle of light and darkness facility, and the temperature was maintained at 25 ± 2 °C with a relative humidity of 55 ± 5%. During the experiment, mice were allowed unrestricted access to food and water and were given 1 week to acclimate. Experimental animals were separated into 6 groups and 8 mice in each group: normal-fat diet group (NFD) with 10% kcal fat (Envigo, Madison, WI, USA), high-fat diet group (HFD) with 60% kcal fat (Envigo, Madison, WI, USA), HFD supplemented with SO (100, 200, and 400 mg/kg) group, and HFD supplemented with GC (200 mg/kg) as a positive control group [24]. For the first two weeks of the experiment, only dietary induction was performed, followed by the 10-week period in which the extract was dissolved in distilled water and given orally daily. The amount of food and water consumed by the animals and their body weight was recorded once a week.

### 2.8. Serum Analysis and Tissue Collection

Indicators for serum analysis were performed as previously described [25]. After the experiment, mice were deprived of food for 12 h and given avertin anesthesia to collect blood samples from their orbital vein. Blood was separated into serum using a centrifuge (Eppendorf, Hamburg, Germany) at 3000× *g* for 20 min at a temperature of 4 °C. Serum analysis was used with an automated clinical chemistry analyzer (DRI-CHEM NX500i; FUJI, Tokyo, Japan), and serum leptin quantification was performed with an ELISA kit (R&D Systems, Minneapolis, MN, USA). Organs were collected and weighed after mice were euthanized. Serum and tissue samples were preserved at −80 °C.

### 2.9. Histological Analysis

Epididymal adipose and liver tissue were immersed in 4% paraformaldehyde for 48 h. Fixed tissues were dehydrated through an automated tissue processor (TP1020; Leica Biosystems, Nussloch, Germany) and embedded in paraffin following previously described methods [26]. Paraffin-embedded blocks were sectioned at 6 µm and dyed with a combination of hematoxylin and eosin (H&E). Observation of tissue staining was conducted under a light microscope, and adipocyte size was measured using Adiposoft software 1.13 (National Institutes of Health, Bethesda, MD, USA).

### 2.10. Histofluorescence Staining

DHE histofluorescence staining was carried out to investigate in situ production of superoxide anion. Briefly, as described previously [27], the tissue slices were balanced in Krebs-HEPES buffer (pH 7.4) for 35 min at 35 °C. Afterward, the sections were soaked in fresh buffer containing 0.01 mM DHE for 2 h at 37 °C. Using an epifluorescent microscope (Carl Zeiss, Göttingen, Germany), digital images of DHE fluorescence were captured with 520–540 nm of wavelength. The images were adjusted to fit a tissue area array of 250 μm^2^. Ethidium fluorescence intensity was quantified utilizing Image J software (version 1.46; National Institutes of Health, Bethesda, MD, USA). The DHE fluorescence intensity ratio was converted into a percentage of the fluorescence intensity in the control group.

### 2.11. Immunohistochemistry

The avidin-biotin complex (ABC) method was utilized for immunohistochemistry. In brief, according to previously described methods [28,29], deparaffinized and hydrated sections were reacted with 0.3% hydrogen peroxide (in 100 mM phosphate-buffered saline, pH 7.4) for 20 min at room temperature. After that, the sections were immersed into 5% normal goat, rabbit, or horse serum (Vector Laboratories Inc., Burlingame, CA, USA) for 30 min at room temperature. Next, the tissue slices were immunoreacted with individual primary antibodies for 48 h at 4 °C: sheep anti-Cu, Zn-superoxide dismutase (SOD1), sheep anti-Mn-superoxide dismutase (SOD2), and mouse anti-4-hydroxy-2-nonenal (4-HNE). The sections undergoing immunoreaction were subsequently incubated with each secondary antibody for 2 h at room temperature: goat anti-sheep IgG and horse anti-mouse IgG (Vector Laboratories Inc., Burlingame, CA, USA). Afterward, the sections were exposed to ABC solution (Vector Laboratories Inc., Burlingame, CA, USA) for 1 h at room temperature. For visualization, the sections were reacted with 0.06% DAB (in 100 mM phosphate-buffered saline, containing 0.1% hydrogen peroxide, pH 7.4). Images of the immunoreactive structures were taken using a light microscope (BX53; Olympus, Tokyo, Japan). These digital images were calibrated into an array of 250 μm^2^ of tissue area. The captured images were converted to 8-bit grayscale with an intensity scale ranging from 0 (black) to 255 (white) to evaluate grayscale intensities. The average optical density of each immunoreactive structure was determined by Image J software. The relative optical density (ROD) was expressed as a percentage of the ROD in the control group.

### 2.12. Western Blot Analysis

Protein was extracted from WAT with RIPA buffer and quantified using the BCA method. Then, 20 μg of protein was separated by 8–10% SDS-PAGE at 100 V for 90 min. Separated proteins were transferred to PVDF membranes by semi-dry transfer cells (Trans-Blot SD Cell; Bio-Rad, Hercules, CA, USA) at 15 V for 30 min. Membranes were blocked with 5% skim milk in TBST for 1 h at room temperature. The membrane was washed 3 times with TBST and incubated overnight at 4 °C with the primary antibody. Afterward, the membrane was rewashed 3 times and incubated with the secondary antibody for 2 h at room temperature. After the incubation, the membrane was washed 3 times, and the specific protein bands were chemiluminescent by ECL solution and visualized with a Western blotting detection system (ImageQuan LAS 500; GE Healthcare, Chicago, IL, USA). The shaded areas of specific bands were quantified using Image J software.

### 2.13. Statistical Analysis

The results were presented as average value ± standard deviation and plotted using GraphPad Prism (version 8.1; GraphPad Software, San Diego, CA, USA). Significance levels were determined by one-way analysis of variance (ANOVA) utilizing SPSS software (version 25.0; Statistical Package for Social Science, Inc., Chicago, IL, USA), and statistical significance was considered at *p* < 0.05.

## 3. Results

### 3.1. Quantification of Quercitrin in SO

**Figure 1:**
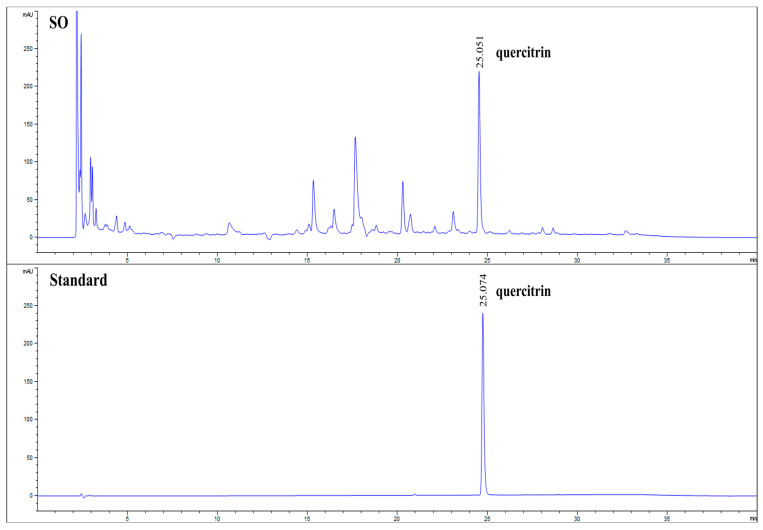
HPLC chromatograms of SO and quercitrin. The identity of quercitrin in SO was determined by comparing the retention times with standards.

The HPLC chromatograms of SO and quercitrin standards are shown in Figure 1. The quercitrin content was determined to be 10.31 ± 0.31 mg/g by comparing the retention time and signal intensity of the peak spectrum of SO and the standard.

### 3.2. SO Has No Apparent Cytotoxicity to 3T3-L1 Cells

**Figure 2:**
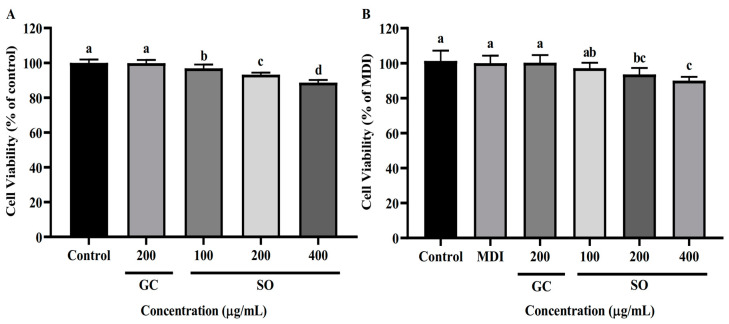
Effect of SO on cell viability of 3T3-L1 preadipocytes (**A**) and adipocytes (**B**). Values are expressed as mean ± SD of experiments (*n* = 3). Different alphabets indicate significant differences at the level of *p* < 0.05.

The cell viability of preadipocytes and adipocytes treated with SO was lower than the control group. However, cell viability was reduced by 11.39% at most in preadipocytes treated with SO for 24 h (Figure 2A) and up to 10.04% in adipocytes treated with SO for 8 days (Figure 2B). The viability of cells treated with all concentrations of SO (100–400 μg/mL) was above 80%, indicating no apparent toxicity to the cells [30].

### 3.3. SO Reduces Lipid Accumulation and ROS Production in 3T3-L1 Adipocytes

**Figure 3:**
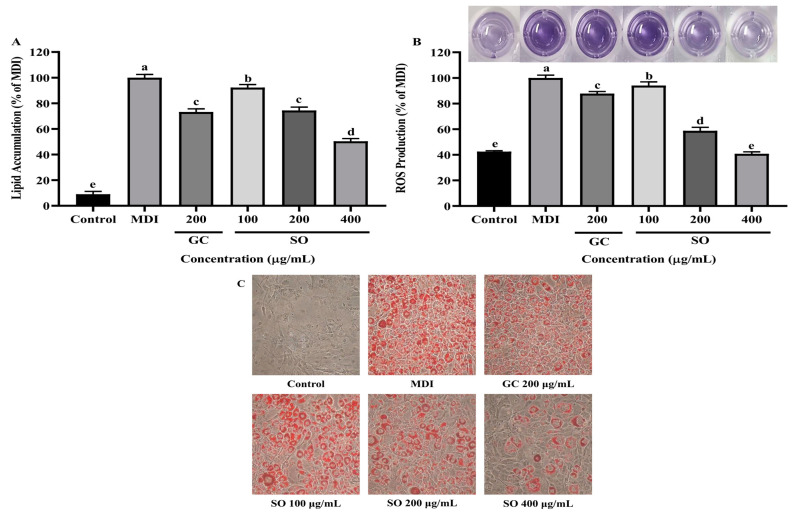
Effect of SO on lipid accumulation (**A**) and ROS production (**B**) in 3T3-L1 adipocytes. (**C**) Oil red O staining on day 8 of adipocyte differentiation. Values are expressed as mean ± SD of experiments (*n* = 3). Different alphabets indicate significant differences at the level of *p* < 0.05.

SO dose-dependently reduced lipid accumulation in 3T3-L1 adipocytes starting from 100 μg/mL (Figure 3A,C). SO showed a similar lipid accumulation inhibition rate to GC at 200 μg/mL and up to 50% lipid accumulation inhibition rate at 400 μg/mL. SO also dose-dependently reduced ROS production in 3T3-L1 adipocytes (Figure 3B). The inhibitory effect of SO on ROS production in 3T3-L1 adipocytes was higher than that of GC at the same concentration (200 μg/mL).

### 3.4. SO Attenuates Body Weight Gain in HFD-Induced Obese Mice without Affecting Appetite

**Figure 4:**
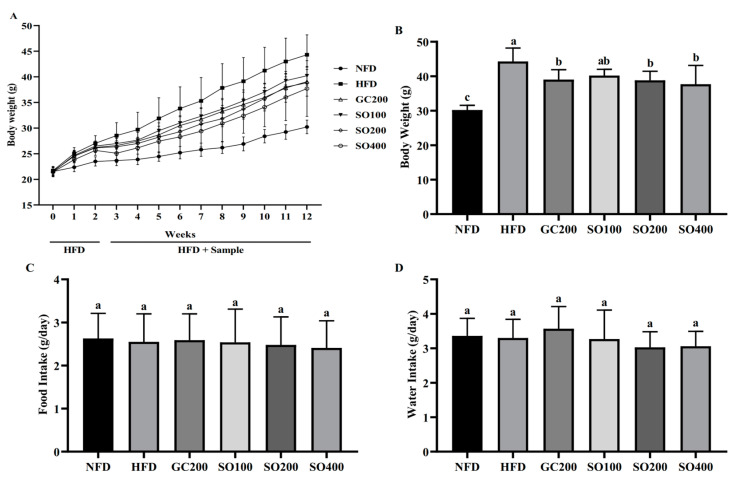
Effects of SO on body weight and diet in HFD-induced obese mice: (**A**) body weight change trend of mice during the experiment; (**B**) final body weight of mice at the end of the experiment; (**C**,**D**) food intake and water intake of mice during the experiment. GC200, HFD supplemented with 200 mg/kg/day of GC; SO100, HFD supplemented with 100 mg/kg/day of SO; SO200, HFD supplemented with 200 mg/kg/day of SO; SO400, HFD supplemented with 400 mg/kg/day of SO. Values are expressed as mean ± SD of experiments. Different letters indicate significant differences at the level of *p* < 0.05.

The average body weight of mice in each group did not differ significantly before diet induction (Figure 4A). Mice in the HFD group (44.31 ± 3.88 g) gained 1.47 times more body weight than the NFD group (30.23 ± 1.33 g) after 12 weeks of diet induction (Figure 4B). Compared to the HFD group, the group supplemented with 100 mg/kg/day (SO100) of SO lost 9.28% of body weight, 12.34% in the 200 mg/kg/day group (SO200), 14.90% in the 400 mg/kg/day group (SO400), and 11.85% in the GC 200 mg/kg/day group (GC200). Body weights of the SO200, SO400, and GC200 groups were statistically different from the HFD group but insignificant between them. Food and water intake were slightly lower in the SO supplementation group but not statistically different (Figure 4C,D).

### 3.5. SO Reduces Adipose Tissue Weight in HFD-Induced Obese Mice

**Figure 5:**
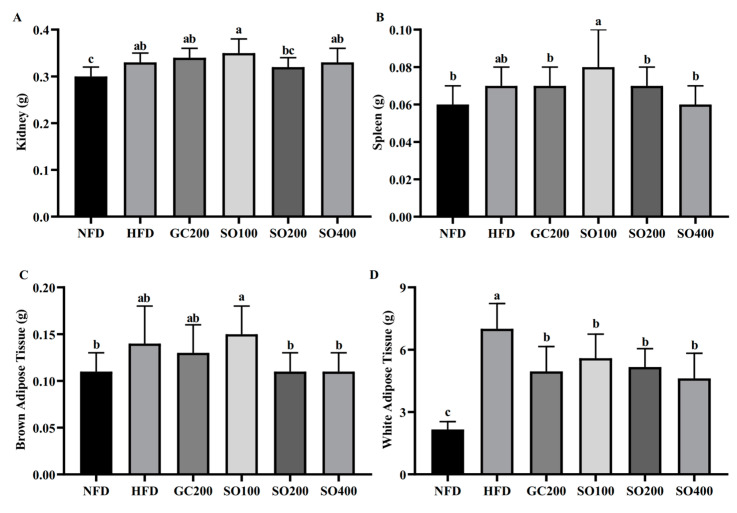
Effect of SO on organ weights in HFD-induced obese mice: (**A**) kidney weight; (**B**) spleen weight; (**C**) brown adipose tissue weight; and (**D**) white adipose tissue weight (visceral, epididymal, perirenal, retroperitoneal, and subcutaneous fat). GC200, HFD supplemented with 200 mg/kg/day of GC; SO100, HFD supplemented with 100 mg/kg/day of SO; SO200, HFD supplemented with 200 mg/kg/day of SO; SO400, HFD supplemented with 400 mg/kg/day of SO. Values are expressed as mean ± SD of experiments. Different letters indicate significant differences at the level of *p* < 0.05.

Kidney, spleen, and BAT weights showed no significant difference between the HFD and SO supplementation groups (Figure 5A–C). SO supplementation significantly reduced WAT (visceral, epididymal, perirenal, retroperitoneal, and subcutaneous fat) weight compared with the HFD group (Figure 5D). Although the reduction in WAT weight was dose-dependently associated with SO, it was not significant.

### 3.6. SO Ameliorates Hepatic Steatosis and Adipocyte Hypertrophy in HFD-Induced Obese Mice

**Figure 6:**
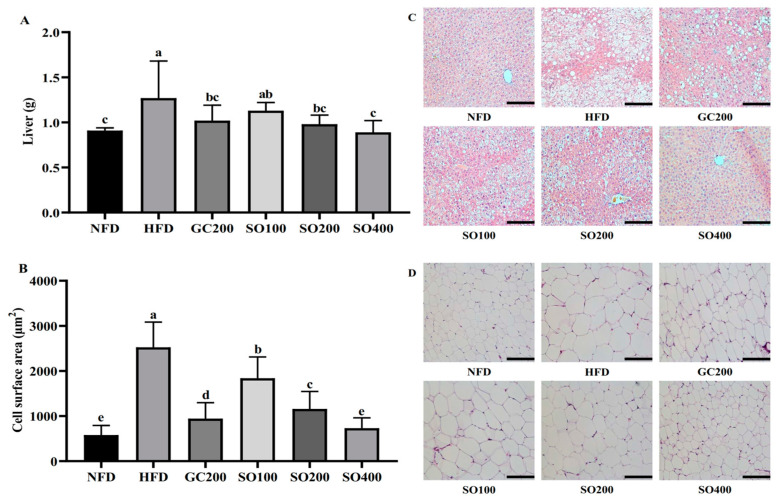
Effects of SO on hepatic steatosis and adipocyte hypertrophy in HFD-induced obese mice: (**A**) liver weight; (**B**) quantification of adipocyte area in white adipose tissue; (**C**) H&E staining of the liver; and (**D**) H&E staining of white adipose tissue. GC200, HFD supplemented with 200 mg/kg/day of GC; SO100, HFD supplemented with 100 mg/kg/day of SO; SO200, HFD supplemented with 200 mg/kg/day of SO; SO400, HFD supplemented with 400 mg/kg/day of SO. The scale bar represents a length of 50 μm. Values are expressed as mean ± SD of experiments. Different letters indicate significant differences at the level of *p* < 0.05.

Liver weight was significantly lower in SO200 and SO400 groups than in the HFD group but insignificant between SO200 and SO400 groups (Figure 6A). Liver H&E staining showed that SO supplementation alleviated the abnormal accumulation of hepatic fat; in particular, the liver tissue of the SO400 group tended toward the NFD group (Figure 6B). H&E staining of epididymal adipose tissue showed hypertrophy of adipocytes induced by the HFD (Figure 6C). SO supplementation reduced adipocyte size in a dose-dependent manner, and the cell size of the SO400 group was similar to the NFD group (Figure 6D).

### 3.7. SO Improves Serum Biochemical Parameters in HFD-Induced Obese Mice

**Figure 7:**
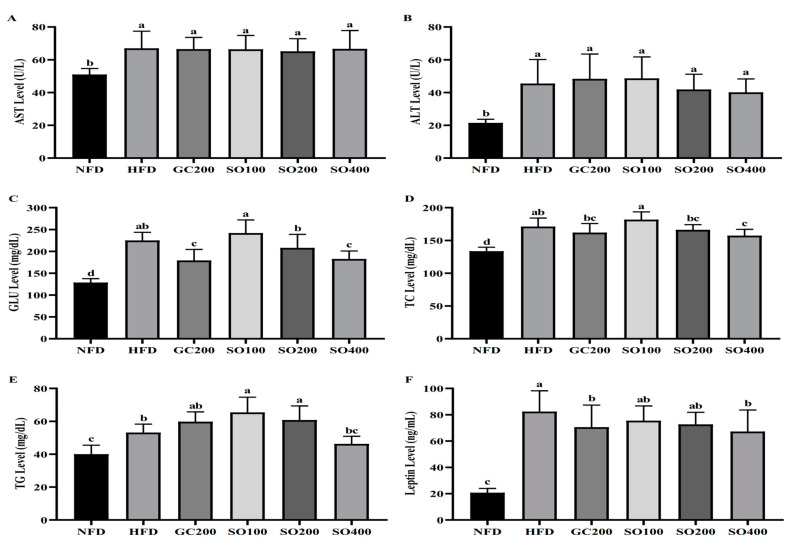
Effects of SO on serum biochemical parameters in HFD-induced obese mice: (**A**) serum aspartate aminotransferase (AST) level; (**B**) serum alanine aminotransferase (ALT) level; (**C**) serum glucose level (GLU) level; (**D**) serum total cholesterol (TC) level; (**E**) serum triglyceride (TG) level; (**F**) serum leptin level. GC200, HFD supplemented with 200 mg/kg/day of GC; SO100, HFD supplemented with 100 mg/kg/day of SO; SO200, HFD supplemented with 200 mg/kg/day of SO; SO400, HFD supplemented with 400 mg/kg/day of SO. Values are expressed as mean ± SD of experiments. Different letters indicate significant differences at the level of *p* < 0.05.

Aspartate aminotransferase (AST) and alanine aminotransferase (ALT) levels were not significantly different between the SO supplementation group and the HFD group (Figure 7A,B). SO supplementation dose-dependently reduced elevated serum glucose (GLU), total cholesterol (TC), TG, and leptin levels due to the HFD, but only the SO400 group was statistically different from the HFD group (Figure 7C–F).

### 3.8. SO Increases WAT Antioxidant Enzyme Expression and Decreases Oxidative Stress in HFD-Induced Obese Mice

**Figure 8:**
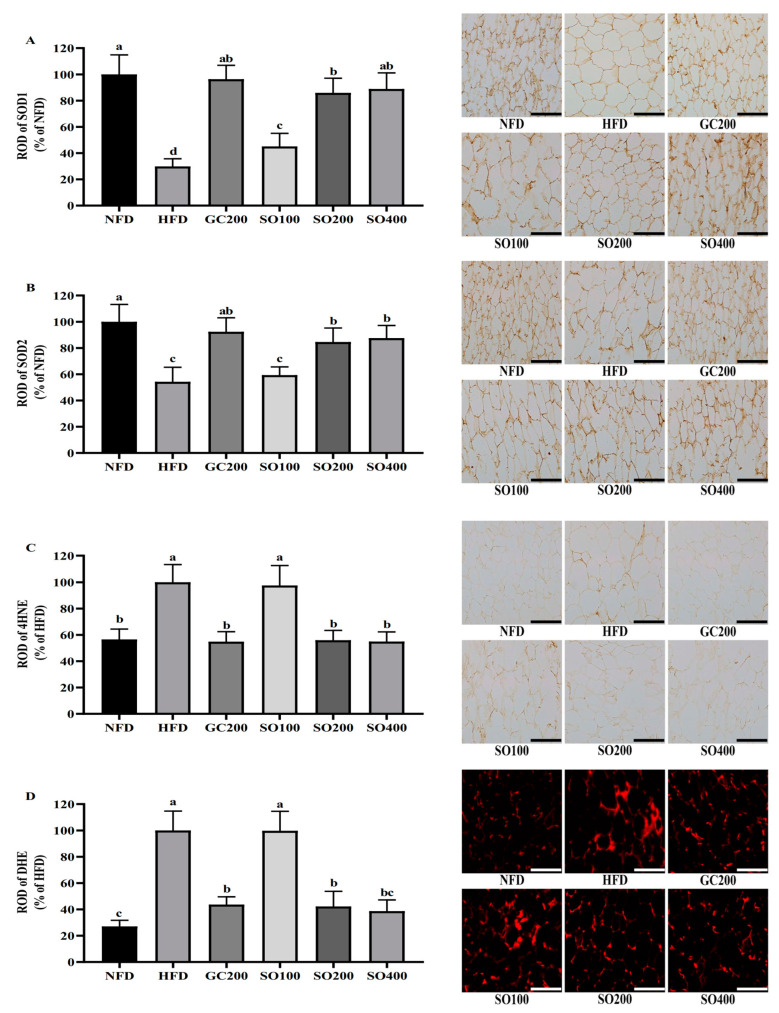
Effect of SO on oxidative stress in WAT of HFD-induced obese mice: (**A**,**B**) SOD1 and SOD2 expression measured by immunohistochemistry in WAT; (**C**) lipid peroxidation products determined by immunohistochemistry of 4HNE in WAT; (**D**) ROS production measured by histofluorescence staining of DHE in WAT. GC200, HFD supplemented with 200 mg/kg/day of GC; SO100, HFD supplemented with 100 mg/kg/day of SO; SO200, HFD supplemented with 200 mg/kg/day of SO; SO400, HFD supplemented with 400 mg/kg/day of SO. The scale bar represents a length of 50 μm. Values are expressed as mean ± SD of experiments. Different letters indicate significant differences at the level of *p* < 0.05.

The expression levels of antioxidant enzymes SOD1 and SOD2 in the HFD group were significantly lower than those in the NFD group by immunohistochemistry on WAT (Figure 8A,B). SO supplementation re-increased the expression of SOD1 and SOD2 but not significantly between SO200 and SO400 groups. SO supplementation above 200 mg/kg reduced ROS production and lipid peroxidation end products as oxidative stress markers (Figure 8C,D). Similar to the expression trend of antioxidant enzymes, there was no significant difference between SO200 and SO400 groups.

### 3.9. SO Improves WAT Energy Metabolism and Promotes Thermogenesis in HFD-Induced Obese Mice

**Figure 9:**
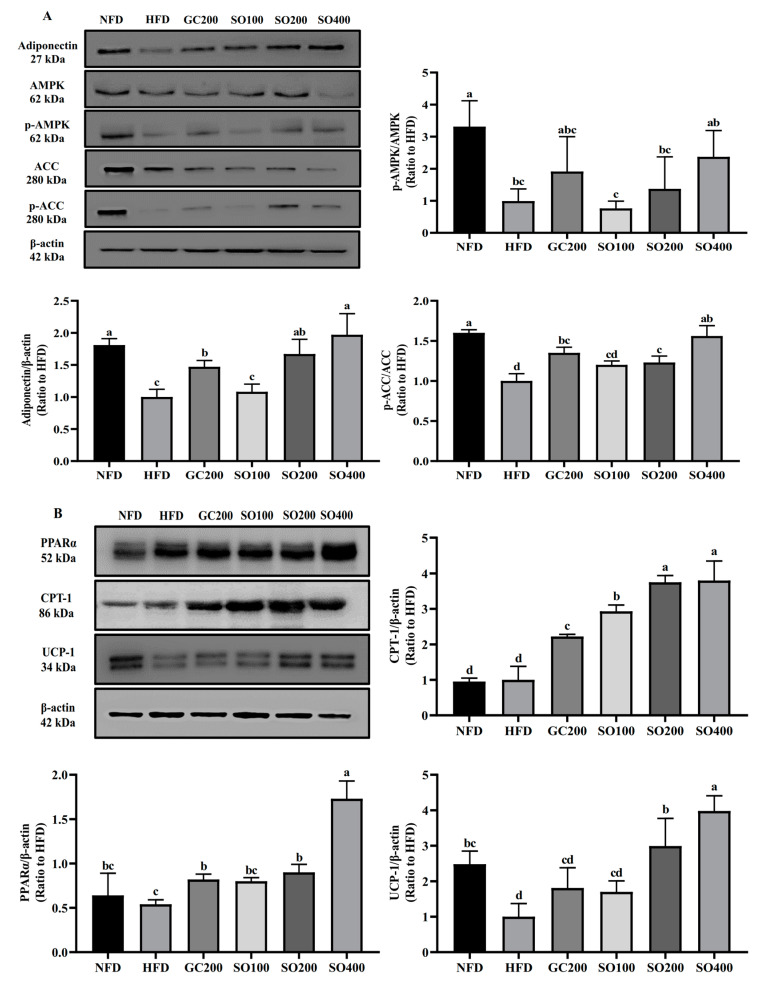
Effects of SO on energy metabolism and thermogenesis in WAT of HFD-induced obese mice: (**A**) adiponectin and AMPK pathways; (**B**) thermogenesis-related factors. GC200, HFD supplemented with 200 mg/kg/day of GC; SO100, HFD supplemented with 100 mg/kg/day of SO; SO200, HFD supplemented with 200 mg/kg/day of SO; SO400, HFD supplemented with 400 mg/kg/day of SO. Values are expressed as mean ± SD of experiments. Different letters indicate significant differences at the level of *p* < 0.05.

Supplementation with SO at doses higher than 200 mg/kg significantly increased adiponectin expression levels and promoted phosphorylation of AMP-activated protein kinase (AMPK) and acetyl-CoA carboxylase (ACC) compared to the HFD group (Figure 9A). SO supplementation above 200 mg/kg also increased the expression of peroxisome proliferator-activated receptor alpha (PPARα) and UCP-1 expression levels in a dose-dependent manner (Figure 9B). In addition, SO dose-dependently increased the expression level of carnitine palmitoyltransferase I (CPT-1), but no further changes were observed at 400 mg/kg.

## 4. Discussion

In a previous study, we isolated and characterized isorhamnetin-3-*O*-d-glucuronide and ellagic acid from SO as potential anti-adipogenic active compounds and validated their lipid accumulation inhibitory effect in 3T3-L1 adipocytes [31]. However, the anti-adipogenic mechanism of SO needs to be clarified, and its effect on obesity-related oxidative stress has been less studied. In this study, we found that SO as a potent antioxidant not only decreased ROS production in 3T3-L1 adipocytes and WAT but also reduced lipid accumulation and cellular hypertrophy, which were associated with the regulation of SO in antioxidant enzymes (SOD1 and SOD2), energy metabolism (AMPK pathway), and thermogenic factors (PPARα, CPT-1, and UCP-1). Furthermore, the administration of SO in HFD-induced obese mice reduced body weight gain, improved hepatic steatosis, lowered serum glucose and lipids, and restored dysregulated adipokines (leptin and adiponectin).

The antioxidant properties of SO are derived from its phenolic acid and flavonoid compounds [32]. In addition to quercitrin, which was used as an index ingredient in this study, the main components in the leaves and stems of SO contained ellagic acid, caffeoylquinic acid, rosmarinic acid, ursolic acid, sanguisorbic acid, catechin, quercetin, kaempferol, sanguisorbigenin, and sanguiin [17,33]. These compounds exhibited antioxidant activity, among which ellagic acid [34], caffeoylquinic acid [35], rosmarinic acid [36], ursolic acid [37], catechin [38], and quercetin [39] have been reported to enhance SOD activity in obese mice. SOD facilitates the transformation of superoxide radicals into H_2_O_2_, which is then converted to water through the assistance of catalase (CAT) and glutathione peroxidase (GPx) [40]. These enzymes collectively establish the antioxidant defense mechanism to counteract free radicals generated by normal mitochondria metabolism [13]. However, excess energy can overload the mitochondria and disrupt the antioxidant defense system [41]. Previous studies have shown that sustained oxidative stress downregulates the expression of thermogenic factors [42]. Although ROS is one of the necessary conditions for mitochondrial thermogenesis [43], respiratory thermogenesis is only possible if the normalized mitochondrial function is ensured. The imbalance of ROS also leads to the ectopic accumulation of TG and the disturbance of energy metabolism caused by mitochondrial dysfunction [44]. The study found that the HFD reduced SOD1 and SOD2 expression in WAT and increased ROS and lipid peroxides (Figure 8A,B). SO supplementation reduced ROS production and oxidative stress by restoring SOD1 and SOD2 expression in WAT to protect mitochondrial function (Figure 8C,D).

Increased ROS in adipocytes leads to the dysregulation of adipokines [10]. In this study, serum leptin levels were notably elevated in the HFD group compared to the NFD group (Figure 7F). The elevated circulating leptin levels can lead to leptin resistance and chronic inflammation [45]. SO supplementation effectively lowered serum leptin levels, although they did not return to normal. Adiponectin is a positively regulated adipokine in adipocytes closely related to insulin sensitivity, skeletal muscle cell glucose uptake, fatty acid β-oxidation in adipocytes, and mitochondrial biogenesis [46]. We observed a significant reduction in adiponectin expression in the WAT of HFD-induced mice (Figure 9A). SO supplementation resulted in increased adiponectin secretion in mice WAT accompanied by decreased serum glucose and lipid levels (Figure 7C–E). These results are similar to previous studies in that the antioxidant capacity of natural products alleviated the oxidative stress increased by an HFD to restore adipokine levels [15].

Adiponectin is also an activator of AMPK and an active ligand of PPARα [47]. AMPK as a regulator of energy homeostasis promotes energy synthesis and suppresses energy expenditure processes, in other words, stimulating lipolysis and inhibiting lipogenesis [48]. We speculated that the AMPK pathway was activated due to the increased adiponectin expression in the WAT of obese mice induced by an HFD (Figure 9A). Activation of AMPK causes direct phosphorylation of downstream ACC to prevent carboxylation of acetyl-CoA to malonyl-CoA [49]. Malonyl-CoA competitively inhibits the activity of CPT-1, which is responsible for facilitating the mitochondrial transmembrane movement of fatty acids (FAs) [50]. SO supplementation enhanced CPT-1 activity and allowed enough FAs to be transported as substrates to mitochondria for β-oxidation and respiratory thermogenesis (Figure 9B).

UCP-1 is responsible for the uncoupling respiratory chain in the inner mitochondrial membrane and allows fatty acid oxidation [51]. When free FAs bind to UCP-1, they cause conformational changes in the activated proteins, leading to the dissipation of the electrochemical gradient in the inner mitochondrial membrane and promoting heat production in the mitochondria [52]. AMPK can promote UCP-1 expression through downstream cascades [53], while PPARα acts as a transcription factor binding to the PPAR-responsive element to facilitate UCP-1 transcription and mitochondrial fatty acid oxidation [54]. Similar to previous studies, SO supplementation did not change the weight of BAT, but the weight of WAT decreased significantly (Figure 5C,D) [55]. This suggests that the SO activates thermogenesis through PPARα and AMPK-induced UCP-1 expression in WAT, thereby attenuating adipocyte hypertrophy and abnormal lipid accumulation.

## 5. Conclusions

Available data suggest that at least 200 mg/kg of SO can reduce ROS production and oxidative stress by increasing antioxidant enzyme expression and improving energy metabolism by activating the AMPK pathway to reduce mitochondrial load. In addition, phosphorylation of AMPK and upregulation of PPARα induced not only UCP-1 expression but also promoted CPT-1 activity to provide FAs as a substrate for oxidative and respiratory thermogenesis. In conclusion, SO improves obesity symptoms and the accompanying oxidative stress without significant toxicity and affecting appetite and is an alternative material for preventing and treating obesity.

## Data Availability

The data presented in this study are available from the corresponding author upon request.
